# Dental Work Needed by 85 per Cent of Draft Recruits

**Published:** 1918-10

**Authors:** 


### Dental Work Needed By 85 Per Cent of Draft Recruits.

New York, Aug. 7.—Eighty-five per cent of the men chosen by the selective draft need dental attention, and the mouths of 60 per cent are in a deplorable condition. These facts were given out yesterday by the Preparedness League of American Dentists from the headquarters of Director General Charles F. Ash, 115 Broadway.

Further statements include the report that 20 per cent of all sick leaves granted to men at the front are due to dental troubles and large numbers of those disabled in France are unfit for military duty because of jaw and mouth conditions.

In view of these facts the Preparedness League of American Dentists is working to save the teeth of American soldiers and sailors and to make their mouths sanitary so that when they arrive over there they will not suffer from abscessed teeth and bad roots.

Inasmuch as there is only one dentist alloted to every 1,000 men in the army the league has been formed to co-operate with draft boards in examining the teeth of selected men and then performing the most necessary operations before the registrants entrain for camp.

It has been estimated that by the work of the league the mouths of American soldiers will be kept clean from diseased teeth to such an extent that the men now in hospitals because of dental infections will be available for service in the trenches.

---

The Southern Medical Association will meet at Ashville, N. C., November 11-14. It is open to all physicians who are members of the county and State medical associations. An interesting program has been prepared, and as Ashville is always an attractive place to visit, it is thought there will be a large attendance despite the fact that so many physicians and surgeons are in war service.

---

Vaccination with a recently discovered serum is being used extensively as a preventive of the contraction of pneumonia from Spanish influenza. The extensive use of this serum is made possible by a million dollar appropriation made by Congress to prevent communicable disease. Physicians connected with the Army Medical School developed the formula for the serum, one treatment with which is said to be sufficient.

